# Liraglutide improved the cognitive function of diabetic mice via the receptor of advanced glycation end products down-regulation

**DOI:** 10.18632/aging.202162

**Published:** 2020-11-26

**Authors:** Haoqiang Zhang, Yafen Chu, Hongwei Zheng, Jing Wang, Bing Song, Yao Sun

**Affiliations:** 1 Department of Endocrinology, First Affiliated Hospital of Jinzhou Medical University, Jinzhou, China; 2 Department of Endocrinology, Affiliated Zhongda Hospital of Southeast University, Nanjing, China; 3 Department of Endocrinology, Ningbo Medical Center Lihuili Hospital, Ningbo, China; 4 Department of Pharmacy, Taikang Xianlin Drum Tower Hospital, Medical School of Nanjing University, Nanjing, China

**Keywords:** liraglutide, cognitive function, diabetes mellitus, receptor of advanced glycation end products

## Abstract

Background and aims Advanced glycation end products (AGEs) and receptor of advanced glycation end products (RAGE), are associated with cognition decline. We aim to investigate the effect of liraglutide on cognitive function in diabetic mice.

Results Diabetic mice showed decreased cognitive function. Moreover, lower glucagon like peptide-1 (GLP-1) levels in plasma were detected in db/db mice. Additionally, up-regulated RAGE and down-regulated glucagon like peptide-1 (GLP-1R) levels were observed in db/db mice. However, decreased GLP-1R and increased RAGE were reversed by liraglutide. We also found decreased cellular activity in cells with AGEs. Moreover, AGEs up-regulated RAGE in PC12 and HT22 cells. However, liraglutide improved the cell activity damaged by AGEs. Although we did not discover the direct-interaction between RAGE and GLP-1R, elevated RAGE levels induced by AGEs were restored by liraglutide.

Conclusion We demonstrated that the cognitive function of diabetic mice was improved by liraglutide via the down-regulation of RAGE.

Methods db/db mice and db/m mice were used in this study. Liraglutide was used to remedy diabetic mice. Neurons and RAGE in hippocampus were shown by immunofluorescence. And then, PC12 cells or HT22 cells with AGEs were treated with liraglutide. GLP-1R and RAGE were measured by western blotting.

## INTRODUCTION

Chronic hyperglycemia is a well-recognized risk factor of cognitive impairment in patients with type 2 diabetes mellitus (T2DM) [1, 2]. Due to the unclear mechanism of diabetic cognition dysfunction, there is no effective treatment for diabetic patients with cognition impairment. It is believed that AGEs formed by non-enzymatic reactions play an essential role in various chronic complications of diabetes [3, 4] including cognitive dysfunction [5, 6]. It was reported that AGEs are involved in aging [5] and dementia [6]. In an interesting animal study, AGEs increased in serum, cortex and hippocampus after injection of galactose, glucose and other reducing sugars. Additionally, the ability of spatial learning and memory of mice was also impaired [7]. It is suggested that AGEs mediate cognitive impairment induced by hyperglycemia. Moreover, increased AGEs levels were found in most of the senile plaques in Alzheimer’s disease (AD), even in the diffuse plaques [8]. It was further confirmed that AGEs modified β-amyloid (Aβ) during its formation [9]. After cross-linking with AGEs, the nucleation and aggregation of Aβ increased in senile plaques [10]. The effects of AGEs depend on its receptor, RAGE. RAGE is a signal-transducing cell surface receptor and promotes Aβ accumulation, which accumulates during AD development [11]. Indeed, not only increased AGEs levels, but also soluble RAGE levels were found in the plasma of diabetic patients with mild cognitive impairment in previous study [12].

Aβ is formed by hydrolysis of its precursor protein (β-APP) by β-site amyloid precursor protein cleaving enzyme 1 (BACE1) [13]. When the ligand binds to RAGE, the formation and aggregation of Aβ in brain were promoted by stimulating BACE1. It is found that RAGE, which is on the blood brain barrier, is an important transporter of Aβ. It transports only Aβ in circulation into the brain, but not out of the brain [14]. More studies have found that RAGE is involved in cell proliferation and apoptosis [15, 16], including neurons [17, 18].

GLP-1 binding to GLP-1R, could stimulate insulin secretion from the pancreatic β-cells and reduces glucagon secretion from the α-cells to keep glycemic homeostasis in the condition of hyperglycemia. It is an endogenous hormone released from L-cells of the intestine after meal [19]. GLP-1 (1–37) peptide is released as a precursor and processed by prohormone convertases. This precursor protein was cleaved and amidated to two GLP-1 active forms, GLP-1 (7–37) and GLP-1 (7–36) [20]. But, these two important peptides had a half-life of only 2 minutes, and degraded by a dipeptidyl-peptidase IV (DPP-4), [21, 22]. This unstable polypeptide regulates the glucose homeostasis of individuals sensitively through this mechanism. However, this limited the use of native GLP-1 in clinical result from unreachable concentration in the blood to the central nervous system (CNS). Although, GLP-1R is widely expressed in the brain, most native GLP-1 was degraded in several minutes. So, the concentration of native GLP-1 is too low to affect CNS directly [23]. GLP-1, produced by the central nervous system, may be involved in cognitive impairment. Indeed, GLP-1 is also produced from the neurons in the solitary tract in the brainstem [24]. Unfortunately, GLP-1 or its receptor is significantly decreased in the brain of even in prediabetic individuals [25].

GLP-1 receptor agonist (GLP-1RA) can avoid degradation and reach an even high blood drug concentration in the blood. Exenatide, extracted from the *saliva* of the *lizard Heloderma suspectum*, shares 53% homology with native GLP-1, and has a half-life of 2.4 hours. Liraglutide is deriving from native GLP-1, share 97% homology with native GLP-1 and with a half-life of 13h [26]. Semaglutide, has a half-life of more than 7 days due to high affinity with albumin [27]. GLP-1RA may play its neuroprotective role not only by lowering plasma glucose, but also play this role directly. GLP-1RA showed its neuroprotective effect by promoting hippocampal progenitor cells proliferation and increasing immature neurons in the hippocampal dentate gyrus [28]. Indeed, long-term usage of liraglutide rescued motor impairment in diabetic db/db mice [29]. Moreover, it can reverse neuronal loss [30]. Additionally, it was showed that liraglutide improved cognition in non-diabetic individuals [31]. Owning to blood-brain barrier, large molecules, like semaglutide cannot reach the CNS, but liraglutide as a small molecule, can directly affect neurons in the brain. Indeed, liraglutide prevented both motor dysfunction in the substantia nigra and basal ganglia. Furthermore, liraglutide induced a marked increase in anti-apoptotic pathways compared to other GLP-1RA [32]. Liraglutide showed effects against hippocampal neurodegeneration in an animal model induced by streptozotocin (STZ). In particular, it improved their ability of learning and memory, and reduced hippocampal neuronal death [33]. In addition, treatment of liraglutide contrasted neuronal damage in the hippocampal in STZ-induced diabetic models [34].

So, we guess that liraglutide may affect the survival of neurons in the central nervous system, especially hippocampal neurons, not only dependent on its hypoglycemic effect, but also may play a neuroprotective role through RAGE. On the one hand, liraglutide may bind to its receptor, and influencing the function of RAGE directly, on the other hand, it may improve the cognition of diabetic mice by influencing the expression of RAGE.

## RESULTS

### GLP-1 decreased in db/db mice after meal

**Figure 1:**
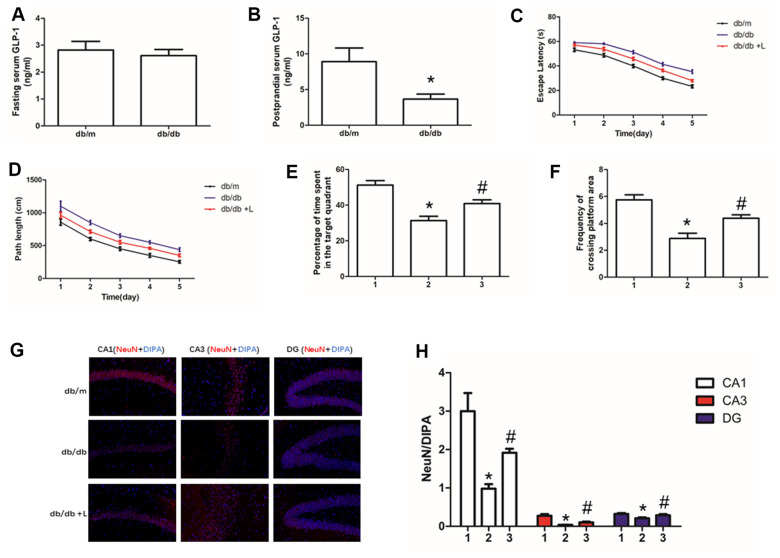
**Liraglutide improved the cognitive function of db/db mice and neuros loss in hippocampus.** "*" significant difference between db/db mice and db/m mice; "#" significant difference between db/db mice with liraglutide and db/db mice without liraglutide; "db/m" of (**A–D, G**) db/m mice without liraglutide; "db/db" of (**A–D, G**) db/db mice without liraglutide; "db/db + L" of (**C**, **D**, **G**) db/db mice with liraglutide at a concentration of 240ug/Kg; "1" of (**E, F, H**) db/m mice without liraglutide; "2" of (**E, F, H**) db/db mice without liraglutide; "3" of (**E, F, H**) db/db mice with liraglutide at a concentration of 240ug/Kg.

In this work, fasting serum GLP-1 level was measured firstly. There was no difference of fasting GLP-1 level between db/db and db/m mice (Figure 1A). However, we detected higher GLP-1 levels in db/m than that in db/db mice after meal (Figure 1B).

### Liraglutide improved cognition decline of db/db mice

Hyperglycemia is one of the most important risk factor of cognition dysfunction in human [1, 2] and animal models [35, 36] with diabetes mellitus. Therefore, we used db/db mice to explore further confirm the conclusion and probe the effect of liraglutide, a plasma glucose lowing-drug on cognitive function of diabetic mice. Indeed, compare to db/m mice without diabetes, db/db mice showed significant cognition decline. Shorter escape latency and path length of healthy mice were recorded than those of diabetic mice (Figure 1C, 1D). In addition, compared with db/db mice, decreased percentage of time spend in the target quadrant and frequency of crossing platform area in db/db mice were found (Figure 1E, 1F). Interestingly, liraglutide significantly improved the cognitive function of diabetic mice. Escape latency, path length, percentage of time spend in platform area and frequency of crossing platform area were restored by liraglutide (Figure 1C–1F).

### Liraglutide increased neurons survival in hippocampus tissue

Neurons are essential for cognition decline. We conducted immunofluorescence of NeuN to detect neurons in the hippocampus of mice. We not only measured decreased neurons in hippocampus of db/db mice, compared with that of db/m mice, but also observed increased hippocampal neurons fluorescence of diabetic mice with liraglutide at CA1, CA3 and DG regions, compared with diabetic mice without liraglutide (Figure 1G, 1H).

### Increased RAGE and decreased GLP-1R in hippocampus were restored by liraglutide

**Figure 2:**
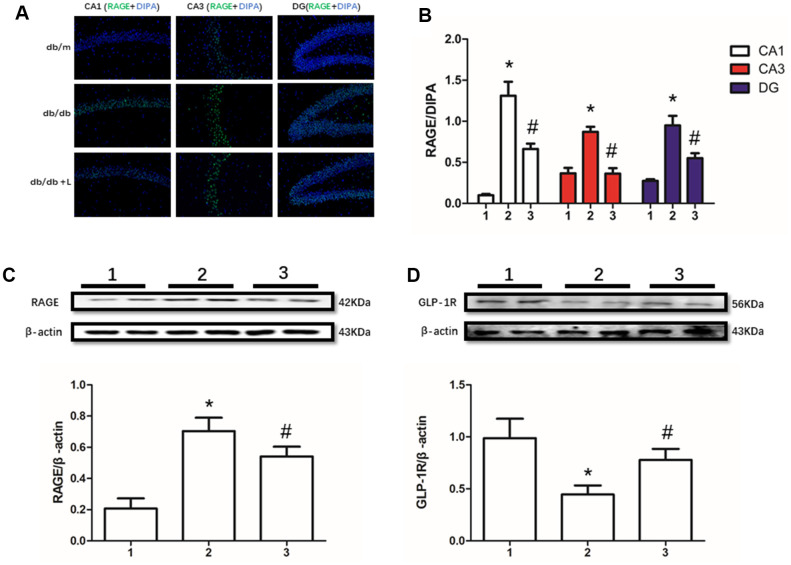
**Liraglutide restored RAGE and GLP-1R of db/db mice in hippocampus.** "*" significant difference between db/db mice and db/m mice; "#" significant difference between db/db mice with liraglutide and db/db mice without liraglutide; "db/m" of (**A**) db/m mice without liraglutide; "db/db" of (**A**) db/db mice without liraglutide; "db/db + L" of (**A**) db/db mice with liraglutide at a concentration of 240ug/Kg; "1" of (**B–D**) db/m mice without liraglutide; "2" of (**B–D**) db/db mice without liraglutide; "3" of (**B–D**) db/db mice with liraglutide at a concentration of 240ug/Kg.

RAGE, including soluble RAGE in serum [12] and local RAGE [37, 38], significantly increases in many diabetic or non-diabetic animal models and human with cognition decline. In this present work, we detected up-regulated RAGE and down-regulated GLP-1R in hippocampus of mice with diabetes compared with non-diabetic mice. However, interestingly, we not only measured restored GLP-1R, but also improved RAGE. Moreover, increased RAGE levels in db/db mice and decreased RAGE levels in db/db mice liraglutide with were detected in CA1, CA3 and DG regions of hippocampus (Figure 2).

### The viabilities of HT22 and PC12 cells were decreased by AGEs

**Figure 3:**
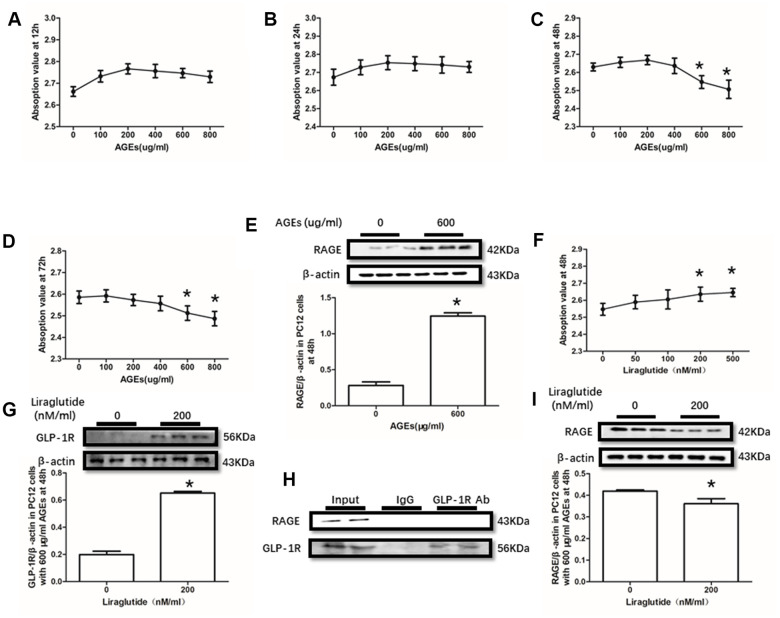
**Liraglutide restored cells viability decline and up-regulated RAGE by AGEs in PC12 cells.** Results in (**A**, **B**) did showed lower cells viability PC12 cells with AGEs at different concentrations than those without AGEs. "*"of (**C**, **D**) cells viability is lower of PC12 cells with AGEs at a concentration of 600ug/ml or 800ug/ml than those without AGEs; "*" of (**E**) significant difference of RAGE levels between PC12 cells with AGEs at a concentration of 600ug/ml; "*" of (**F**) cells viability is higher of PC12 cells with AGEs at a concentration of 600ug/ml and liraglutide at a concentration of 200nM/ml than those with AGEs at a concentration of 600ug/ml but without liraglutide. "*" of (**G**, **I**) significant difference of RAGE levels between PC12 cells (with AGEs at a concentration of 600ug/ml) with liraglutide at a concentration of 200nM/ml and those without liraglutide. Results in (**H**) did not showed the direct interaction between GLP-1R and RAGE in PC122 cells.

**Figure 4:**
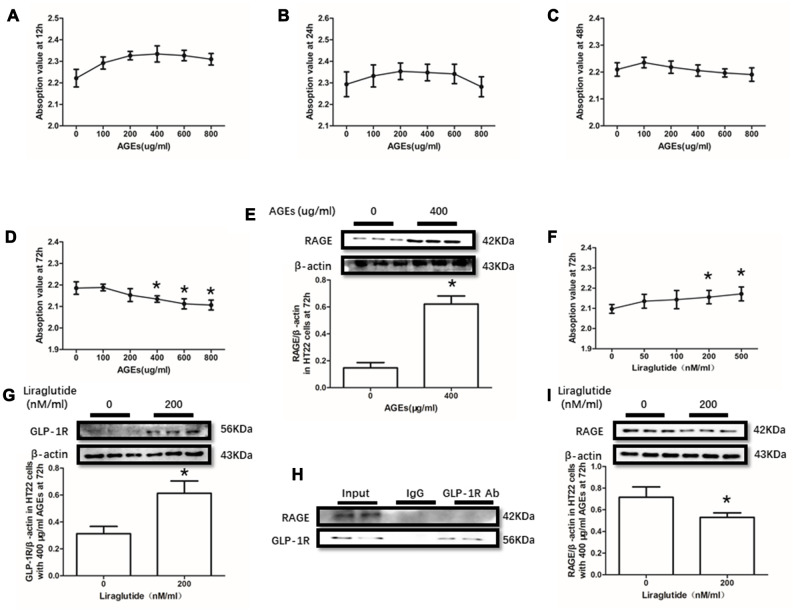
**Liraglutide restored cells viability decline and up-regulated RAGE by AGEs in HT22 cells.** Results in (**A**–**C**) did showed lower cells viability HT22 cells with AGEs at different concentrations than those without AGEs. "*"of (**D**) cells viability is lower of HT22 cells with AGEs at a concentration of 400ug/ml, 600ug/ml or 800ug/ml than those without AGEs; "*" of (**E**) significant difference of RAGE levels between HT22 cells with AGEs at a concentration of 400ug/ml; "*" of (**F**) cells viability is higher of HT22 cells with AGEs at a concentration of 400ug/ml and liraglutide at a concentration of 200nM/ml than those with AGEs at a concentration of 400ug/ml but without liraglutide. "*" of (**G**, **I**) significant difference of RAGE levels between HT22 cells (with AGEs at a concentration of 400ug/ml) with liraglutide at a concentration of 200nM/ml and those without liraglutide. Results in (**H**) did not showed the direct interaction between GLP-1R and RAGE in HT22 cells.

AGEs levels increased in diabetic human. Additionally, AGEs levels are associated with many its complications including cognitive dysfunction [3, 4, 6]. So, AGEs peptide was used to induce cells viability declines in this study. Unfortunately, AGEs failed to induce declined viability of PC12 cells at a concentration of 100μg/ml, 200μg/ml or 400μg/ml (Figure 3A–3D). Nevertheless, AGEs (600μg/ml and 800μg/ml) increased PC12 cells viability after administration at both 48h and 72h (Figure 3C, 3D). Moreover, although AGEs administration did not significantly cause HT22 cells viability at 12h, 24h and 48h (Figure 4A–4C), decreased viability of HT22 cells with AGEs at a concentration of 400μg/ml, 600μg/ml or 800μg/ml was measured at 72h (Figure 4D). Not surprisingly, up-regulated RAGE at 48h in PC12 cells with AGEs (600μg/ml) and at 72h in HT22 cells with AGEs (400μg/ml) (Figures 3E, 4E).

### The viabilities of HT22 and PC12 cells were improved by liraglutide

GLP-1RA showed obvious neuroprotective effect in neuronal cell lines [39, 40], animal models [31, 41] with or without diabetes mellitus and diabetic patients [42]. In this recent study, improved viability of PC12 cells with by AGEs (600μg/ml) at 48h and HT22 cells with by AGEs (400μg/ml) at 72h by liraglutide at a concentration of 200nM or 500nM were measured (Figures 3F, 4F). Moreover, increased GLP-1R induced by liraglutide (200nM) was detected (Figures 3G, 4G).

### GLP-1R did not pull-down RAGE

As GLP-1R and RAGE are proteins insert in the cell membrane, we conducted a co-immunoprecipitation assay to investigate the direct interaction between GLP-1R and RAGE. However, we did observe RAGE in the mixture pulled-down by GLP-1R antibody both in PC12 cells and HT22 cells (Figures 3H, 4H).

### Liraglutide down-regulated RAGE expression

Despite no direct interaction was measured between GLP-R and RAGE by co-immunoprecipitation assay, we found decreased GLP-1R in PC12 cells and HT22 cells with liraglutide (200nM) at 48h or 72h respectively (Figure 3I, 4I).

## DISCUSSION

GLP-1 showed its neuroprotective effects in several previous studies. Lower GLP-1 levels were detected not only in the serum of T2DM patients but also in obesity individuals without diabetes [43, 44]. Additionally, decreased GLP-1R levels in obese or diabetic mice with cognitive impairment in multiple tissue, including brain were observed [25]. Moreover, the cognitive function of these rodents was restored after overexpression of GLP-1R by transgenic technology [45]. Last but not least, GLP-1RA improved the cognitive function of patients with diabetes, and even of non-diabetic patients with degenerative neuropathy and cerebral infarction [46, 47]. So, in this present study, GLP-1 levels were monitored in serum of mice with or without diabetes. Although, there was no significant fasting serum GLP-1 decreased in db/db mice with diabetes, compared with db/m mice without hyperglycemia, we measured lower postprandial serum GLP-1 in diabetic mice than that in non-diabetic mice.

As lower GLP-1 were measured in diabetic mice, liraglutide, a small molecular GLP-1RA, that could cross BBB and cannot be inactivated by DPP-4 was used to remedy diabetic cognition dysfunction. Interestingly, fewer time of escape latency, as well as shorter path length, percentage of time spend in platform area and frequency of crossing platform area of mice with liraglutide treatment were monitored. Neurons loss is an important sign of cognition dysfunction, in the animal experiment, less neurons were detected by immunofluorescence. Additionally, compared to db/db mice without liraglutide, we measured increased neurons in the hippocampus (including CA1, CA3 and DG regions) of db/db mice with liraglutide.

Despite the neuroprotective effect of liraglutide was confirmed in this study, however, the mechanism underlies remains unclear. AGEs is associated with multiple diabetic complications including degenerative neuropathy of the brain [12]. AGEs is not only promoting cognition decline by its cross-link with Aβ [8–10], but also binding to RAGE to influence the transportation of Aβ. In a recent study, it was reported that RAGE only transports Aβ in circulation into the brain, but cannot carry it out [14]. Indeed, in a previously study, association between soluble RAGE and cognition function was found [12]. In this work, increased RAGE and decreased GLP-1R in diabetic mice were recovered by liraglutide *in vivo*. Due to the role of liraglutide is an anti-diabetic drug, the reason of its effect on cognitive function need to be further explored. We designed the following cells study with the same concentration of glucose to confirm the role of decreased RAGE result from liraglutide administration involved in cognition, and to exclude its hypoglycemic effect only.

In the *in vitro* study, AGEs not only induced elevated RAGE, but also decreased cells viabilities in both PC12 cells and HT22 cells. So, we guess that AGEs induced cells viability declines probably result from changes of RAGE. To further demonstrate the neuroprotective effect of liraglutide, it was used to remedy the viability decline of PC12 and HT22 cells. AGEs induced neurons loss was improved by liraglutide, with an elevated GLP-1R level detected in PC12 and HT22 cells.

GLP-1R and RAGE are both G-protein coupled receptor and inserted in the membrane. Although several researchers suggested that GLP-1 administration may down-regulate the level of RAGE [48, 49], the direct interaction between GLP-1R and RAGE remains need more studies. Here, we conduction a co-immunoprecipitation assay to investigate the direct interaction between GLP-1R and RAGE. Unfortunately, RAGE was not being detected in the mixture pulled-down by GLP-1R antibody both in PC12 cells and HT22 cells. However, down-regulated RAGE by liraglutide was measured in the above cell lines.

## CONCLUSIONS

In general, liraglutide improved the cognitive function of db/db mice and restored their neurons loss in hippocampus. This neuroprotective effect may partly result from the down-regulated RAGE, rather than its hyperglycemic effect only or the direct interaction between GLP-1R and RAGE. However, the mechanism of RAGE regulation by GLP-1R and downstream signaling pathway need be further studied.

## MATERIALS AND METHODS

### Animal housing and treatment

Male db/db and db/m mice were purchased from Beijing HFK Biotechnology Co., Ltd. (Beijing, China) and housed in a specific pathogen-free animal center. All mice were fed with their specific diet and bacteria free water. 7-week-old db/db mice were randomly divided into 2 groups: db/db group (N=8) and db/db+L group (N=8) after adaptive feeding for 1 week. Mice in the db/db+L group were given liraglutide at a dose of 240μg/kg/day [50] by subcutaneous injection for 16 weeks, while the age matched db/m group (N=8) and db/db group were administered with the vehicle at the same volume. At the end of the experiment, all mice were anesthetized by 4% halothane anesthesia and sacrificed by cervical vertebra dislocation. Blood and brain tissue were fresh collected and stored in -80° C for further research. The study was conducted in accordance with the Guide for the Care and Use of Laboratory Animals and approved by the Animal Studies Committee of our institution.

### ELISA assay

Blood samples (50μl for each time) were collected after fasting for 8h and intragastric administration of glucose (2g/kg) for 30minutes respectively from heart. Fasting and postprandial serum GLP-1 levels were carried out according to the Manufacturer's instructions (CUSABIO, Wuhan, China, Catalogue No.: CSB-E08118m).

### Water maze

Morris water maze tests were conducted to detect the cognitive function after the above experiments as our previous study [51]. On the first day of the test, mice were allowed to swim freely to adapt the environment for 1 min without the platform. And then, each mouse was trained for five consecutive days (4 times/day with 3 intervals for 20 min) in the test with platform. The time of each mouse took to find the platform (escape latency) and the total length of the path (path length) were recorded. The frequency of crossing the platform area and the percentage of time spent in the target quadrant were also recorded.

### Cells culture and treatment

HT22 and PC12 cells were purchased from American Type Culture Collection (ATCC, Rockville, MD). PC12 cells were cultured in Dulbecco’s modified eagle’s medium (DMEM) containing glucose (25mM) and supplemented with fetal bovine serum (FBS) (10%), penicillin (100U/ml), streptomycin (100μg/ml) and horse serum (5%) in an incubator at 37° C with 5% CO2. Cells were harvested for passaging when plates were 90% confluent. HT22 cells were cultured in the same medium without horse serum. Additionally, PC12 cells differentiation was administrated with nerve growth factor [52, 53] (50ng/ml). PC12 cells were stimulated to form a sympathetic neuron-like phenotype with neurite outgrowth for 3d. Then, cells were collected for further use.

### 3-(4,5-dimethylthiazol-2-yl)-2,5-diphenyltetrazolium bromide (MTT) assay

For AGEs stimulating, cells (5×10^3^) in 100 μl of DMEM were added to 96-well plates with different concentrations of AGEs (0μg/ml, 100μg/ml, 200μg/ml, 400μg/ml, 600μg/ml and 800μg/ml) for 12, 24, 48and 72h respectively. Liraglutide administration was performed with concentrations of 0nM, 50nM, 100nM, 200nM, 500nM in the medium with cells induced by AGEs for 48 or 72h. Then, MTT (20μl per well) with a concentration of 5mg/mL in phosphate-buffered saline was added and the plates were cultured in an incubator with 5% CO2 at 37° C for 4h. After medium with MTT was removed, dimethyl sulfoxide (DMSO) was added with a volume 150μl per well. Absorbance was measured by a microplate reader at 490 nm.

### Immunofluorescence

Fresh brains of mice were collected and embedded with OTC to get frozen section with hippocampus. Before being incubated with rabbit-anti-mouse primary antibody to NeuN (Bioss, Beijing, China, Catalogue No.: bs-10394R) or RAGE (Bioss, Beijing, China, Catalogue No.: bs-4999R) at 4° C overnight, all slices on the glasses were repaired in the high-pressure cooker by citrate sodium and blocked in PBS containing 5% normal goat serum for 1h at room temperature. After washing with PBS 3 times, Alexa Fluor 594 (Bioss, Beijing, China, Catalogue No.: bs-0295G-AF594) or FITC (Bioss, Beijing, China, Catalogue No.: bs-0295G-FITC) conjugated goat-anti-rabbit secondary antibody were used for detection of primary antibodies in dark environment. After sections were washed for 3 times with PBS, NeuN and RAGE were observed by a fluorescence microscope.

**Western blotting**

Western blotting carried out according to our previously described protocol [54] and described briefly as follows. Hippocampus tissue and above cells were isolated or collected for protein extraction by radioimmunoprecipitation (Wanlei biotechnology co. Ltd, Shenyang, China, Catalogue No.: WLA016a) and measured by a BCA assay according to the Manufacturer's instructions (Wanlei biotechnology co. Ltd, Shenyang, China, Catalogue No.: WLA004b). Proteins were separated in SDS - PAGE gels, and transferred to polyvinylidene fluoride membranes. Rabbit-anti-mouse primary antibodies were used to bind target proteins including RAGE (Bioss, Beijing, China, Catalogue No.: bs-4999R) and GLP-1R (SANTA CRUZ BIOTECHNOLOGY, INC., Dallas, Texas, USA, Catalogue No.: sc-390773) at 4° C overnight. And then, goat-anti-rabbit secondary antibody conjugated with HRP (Wanlei biotechnology co. Ltd, Shenyang, China, Catalogue No.: WLA023a) incubation was performed to binding primary antibodies. ECL kit (Wanlei biotechnology co. Ltd, Shenyang, China, Catalogue No.: WLA006a) was utilized before exposure to detect the protein levels. All experiments were repeated at least 3 times.

### Co-immunoprecipitation assay

Co-immunoprecipitation assay was performed with a kit (Wanlei biotechnology co. Ltd, Shenyang, China, Catalogue No.: WLA112a) according to the manufacturer's protocol. Briefly, PC12 or HT22 cells in culture dishes (100×100 mm) (90% covered) were harvested and dissolve with specified lysis buffer in the kit described above. Before prepared protein A beads were used to capture the complex including GLP-1R, primary antibody of anti-GLP-1R was incubated with measured total protein. Complex with beads was boiled. After centrifugation, samples were got for further western blotting to detect RAGE.

### Statistical analysis

All data were described as mean ± standard deviation. Statistical differences were determined by using Student’s t-tests, and one-way ANOVA followed by LSD for multiple-comparison tests. Data were analysis by SPSS 22.0 (SPSS Inc., Chicago, IL, USA). P<0.05 was considered as significant difference.
